# Developing HPV Vaccination Communication Strategies: Assessing Knowledge, Attitudes, and Barriers Among Healthcare Professionals in Kazakhstan

**DOI:** 10.3390/vaccines12111225

**Published:** 2024-10-28

**Authors:** Fatima Kassymbekova, Alexander Rommel, Dilyara Kaidarova, Ardak Auyezova, Saule Nukusheva, Gauhar Dunenova, Raikhan Bolatbekova, Indira Zhetpisbayeva, Gulzada Abdushukurova, Natalya Glushkova

**Affiliations:** 1 Department of Public Health and Social Sciences, Kazakhstan’s Medical University “KSPH”, Almaty 050060, Kazakhstan; ardak.auyezova@gmail.com; 2 Department of Epidemiology and Health Monitoring, Robert Koch-Institute, D-12101 Berlin, Germany; rommela@rki.de; 3 Kazakh Institute of Oncology and Radiology, Almaty 050000, Kazakhstan; dilyara.kaidarova@gmail.com (D.K.); r.bolatbekova@gmail.com (R.B.); 4 Department of Health Policy and Management, Asfendiyarov Kazakh National Medical University, Almaty 050000, Kazakhstan; nukusheva.s@kaznmu.kz; 5 Department of Epidemiology, Biostatistics and Evidence Based Medicine, Al-Farabi Kazakh National University, Almaty 050040, Kazakhstan; gauhar.dunenova@gmail.com; 6 Department of General Medical Practice-2, Asfendiyarov Kazakh National Medical University, Almaty 050000, Kazakhstan; zhetpisbayeva.i@kaznmu.kz; 7 Department of Therapy, Shymkent Institute of Postgraduate Studies, Akhmet Yassawi University, Shymkent 160013, Kazakhstan; gulzada.abdushukurova@ayu.edu.kz; 8 Health Research Institute, Al-Farabi Kazakh National University, Almaty 050040, Kazakhstan; glushkovanatalyae@gmail.com

**Keywords:** HPV, HPV vaccine uptake, healthcare professional, knowledge, awareness attitudes, perception

## Abstract

Background: Cervical cancer, predominantly caused by human papillomavirus, remains a major public health issue globally and in Kazakhstan, where it ranks among the most common cancers in women. A pilot HPV vaccination programme in Kazakhstan was suspended in 2017 due to mass parental refusals, and it is planned to be restarted in the coming years. This study aims to assess the knowledge, attitudes, barriers, and sources of information about HPV and the HPV vaccine among healthcare professionals in Kazakhstan. Methods: A cross-sectional study (December 2022–May 2023) involving 1189 healthcare professionals in Kazakhstan used a self-administered questionnaire. Statistical analysis included descriptive statistics, mean knowledge score, between-group comparisons, and binary logistic regression to identify factors linked to higher knowledge and vaccine recommendation. Results: The study found that the average knowledge score for HPV and the HPV vaccine among participants was 11 out of a possible 18. Correct answers to the questionnaire were observed more frequently among physicians than among nurses (*p* < 0.001). In our study, 72.6% of healthcare professionals expressed a positive intention to recommend the HPV vaccine. The likelihood of recommending the HPV vaccine was significantly higher among those with higher knowledge of HPV and its vaccine (OR 1.8; 95% CI 1.3–2.5; *p* < 0.001), those familiar with cervical cancer patients (OR 2.0; 95% CI 1.5–2.8; *p* < 0.001), and those with positive attitudes towards the COVID-19 vaccine and childhood vaccination (OR 2.3 and 1.5, respectively). Healthcare professionals identified key barriers to HPV vaccination, including public mistrust (49.4%), fear of side effects (45.9%), and insufficient knowledge among healthcare professionals themselves (30.3%). Information from the internet, including articles and journals, was the most commonly used source of information, followed by social media and colleagues. Conclusions: The disparities identified call for a tailored, multifaceted communication strategy that addresses the diverse needs of health professionals to address the differences in awareness between different groups, in order to ensure successful implementation and coverage of HPV vaccination across Kazakhstan.

## 1. Introduction

Cervical cancer (CC) was the fourth most common cancer among women, with an estimated 604,127 cases and 341,831 deaths worldwide in 2020, mostly in developing countries due to limited access to early detection and prevention [1]. In Kazakhstan, CC ranks second among all cancers in women, with an age-standardised incidence of 15.7 and a mortality rate of 7.2 per 100,000 women. [2]. Persistent infection with high-risk human papillomavirus types (HR-HPV) is a major factor associated with the development of CC and oropharyngeal, anal, penile, vaginal, and vulvar malignancies in women and men. HPV-associated cancers account for 5.2% of all cancers worldwide and an average of 8.0 per 100,000 person-years, with up to 80% of cases attributable to cervical cancer [3]. According to GLOBOCAN, the incidence of HPV-associated cancer types in Kazakhstan in 2020 included more than 3300 new cases [4]. In addition to malignant neoplasms, HPV types with low oncogenic risk cause benign diseases such as condylomatosis, and papillomatosis of the genital, oropharyngeal, and laryngeal areas in adults and children, significantly affecting health and quality of life and increasing costs for patients and the healthcare system. The prevalence of HPV varies between regions and populations, with a global average prevalence of 32.1% [5]. In Kazakhstan, the prevalence of HPV infection among women is significant, ranging from 39.1% to 55.8%, with one of the most common types being HPV 16 [6,7]. The prevalence of HPV in men worldwide is also high, at 31% for any HPV and 21% for HR-HPV [8].

Primary prevention of HPV-associated disease has been possible since the introduction of the first HPV vaccine in 2006 [9]. HPV vaccination, initially recommended for girls, has been extended to boys in several countries. Implementing a universal, gender-neutral vaccination strategy strengthens herd immunity, advances the goal of eliminating cervical cancer, prevents HPV-related cancers in men, promotes gender equality, and reduces stigma and misinformation associated with the vaccine [10]. Cervical screening and HPV vaccination, combined with timely treatment, make cervical cancer almost completely preventable [11]. In 2013, a pilot, school-based HPV vaccination programme for girls aged 12–15 years in four regions was launched in Kazakhstan, but it was discontinued in 2017 [12]. The Ministry of Health of Kazakhstan has announced the resumption of HPV vaccination in 2024 [13].

HPV vaccination coverage varies worldwide but is considered to be suboptimal [14]. There are many types of barriers to vaccine uptake, including socioeconomic and communication-related factors. Barriers can arise from the attitudes and actions of governments, health professionals and organisations, schools and parents, guardians, and adolescents themselves, and the importance of specific barriers varies from country to country. One of the main barriers to vaccination is low awareness, misconceptions, and lack of information [15]. Myths surround the vaccine because of its novelty and association with the reproductive system [16,17].

Healthcare providers play a key role in advising parents about their children’s immunisation, as they are considered valid and reliable sources of information. Strong recommendation from healthcare providers can increase vaccine uptake by three to nine times [18]. However, misconceptions and lack of knowledge about HPV among healthcare professionals can significantly hinder vaccine uptake [19]. In addition to knowledge gaps, healthcare providers have identified children’s age, time constraints, cost, and insurance coverage as significant barriers to recommending HPV vaccination [20,21].

Given the high incidence and prevalence of HPV infection and cervical cancer in Kazakhstan, and the critical role of health professionals, the overall aim of this study was to identify barriers to and facilitators of HPV vaccine uptake in the health sector. Specific objectives were to assess the level of knowledge about HPV and the HPV vaccine and the intention to recommend the HPV vaccine, in order to draw conclusions on the existing barriers to the introduction and implementation of HPV vaccination among health professionals in the Republic of Kazakhstan.

## 2. Materials and Methods

This study was part of a larger project investigating attitudes to HPV vaccination among different populations, including parents and guardians, health professionals, and teachers. The full study protocol has been published elsewhere [22]. The present sub-study was conducted from December 2022 to May 2023 in different regions of the Republic of Kazakhstan as a survey among practicing health professionals with secondary (nurses) and higher (physicians) education and different specialisations, using a validated online questionnaire. In Kazakhstan, nurses have various specialisations, including general nursing, midwifery, emergency, pharmacy and dental nursing, massage therapy, and others. Similar to physicians, they are categorized according to their respective specialty. The link to the survey was distributed to professional communities through instant messengers and social networks, as well as to medical organisations through local state authorities.

### 2.1. Tools and Measurements

The questionnaire designed for health professionals included common general and specific sections. The common questionnaire for all target groups (parents/guardians, health professionals, and teachers) included questions on sociodemographic characteristics such as age, place of residence, gender, and income. The specific section for health professionals included questions on professional characteristics (work experience, place of employment, specialisation), knowledge about HPV and HPV vaccination, and willingness to recommend the HPV vaccine. There were a total of 18 knowledge questions, presented in detail in the study protocol [22], which were scored as follows: correct answers or existing knowledge scored 1 point, and incorrect answers or “I don’t know” scored 0. One of the survey questions asked, “How many doses of the HPV vaccine are needed for girls under 15 years of age?” Initially, three doses were recommended, but the WHO later revised this to two, and in 2022, the WHO Strategic Advisory Group of Experts on Immunization endorsed a single-dose option to increase global coverage. In our questionnaire, both 1 and 2 doses were accepted as correct answers [23]. Thus, the maximum score for all answers was 18 points. The questionnaire also included questions to assess the time since knowledge of HPV and HPV vaccination had increased, and where this knowledge had been acquired. Questions were also asked about whether health professionals recommended the HPV vaccine, and about their attitudes towards the COVID-19 vaccine and towards vaccines included in the national vaccination schedule.

The sample size for the study was calculated using the following formula

n = 100 + 50i,

where i is the number of independent variables in the final logistic regression model [24]. The number of independent variables in our model is 14, so the sample size according to this formula is 800 people.

### 2.2. Statistical Analysis

All statistical analyses were performed using SPSS 24.0 software (IBM Corp., New York, NY, USA). Descriptive analyses were used to describe sociodemographic variables, knowledge, intentions, and barriers to HPV vaccination, and data regarding HPV vaccination communication. Normality tests showed that the total knowledge scores were not normally distributed, so the Mann–Whitney U test and the Kruskal–Wallis test were used to compare the median and interquartile range (IQR) of knowledge scores across groups. Pearson’s chi-squared test was used to compare the proportion of correct answers and sources of information between participants within the ‘nurses vs. physicians’ and ‘low knowledge vs. high knowledge’ groups. Low-knowledge health professionals were defined as those who scored less than 11 on all knowledge assessment questions, whereas high-knowledge health professionals were defined as those who scored 11 or more. Binary logistic regression was used to assess the association between independent sociodemographic and professional correlates and the dependent variables of health professionals’ knowledge of HPV and the HPV vaccine and intention to recommend HPV vaccination. Odds ratios (ORs) with 95% confidence intervals (CIs) were calculated, and a *p*-value < 0.05 was considered statistically significant. This study was approved by the Ethics Committee of the Kazakhstan’s Medical University “KSPH” (No. 138 of 31 May 2021).

## 3. Results

### 3.1. Sociodemographic Characteristics and Knowledge Scores for HPV and HPV Vaccination

A total of 1230 responses were received, of which 40 were excluded because they did not meet the inclusion criteria (37 did not work as health professionals, 3 did not meet citizenship requirements). This left 1189 responses from health professionals, both physicians and nurses, for analysis. The median age of the respondents was 37.0 years (IQR28.0–48.0). The detailed characteristics of the respondents, including their professional characteristics, are shown in Table 1, as are the median scores for knowledge of HPV and the HPV vaccine in the groups.

### 3.2. Knowledge of HPV and HPV Vaccine Among Healthcare Professionals

The median score for knowledge of HPV and the HPV vaccine among healthcare professionals in Kazakhstan was 11.0 [7.0–14.0] points (median, IQR) out of a maximum of 18 points. A high level of knowledge (defined as a percentage of correct responses of over 80%) was demonstrated by 17.1% of healthcare professionals. Physicians had significantly higher knowledge scores than nurses (13.0 vs. 9.0, *p* < 0.001), and primary care workers outperformed hospital staff (11.0 vs. 10.0, *p* < 0.001). Healthcare professionals specialising in obstetrics and gynaecology had the highest knowledge (14.0), followed by those specialised in general practice and other specialties (10.0) (*p* < 0.001). Knowledge increased with experience, and urban respondents scored higher than rural respondents (11.0 vs. 10.0, *p* < 0.001). A strong association was found between higher knowledge and the intention to recommend HPV vaccination, indicating that better informed professionals are more likely to support vaccination (Table 1).

**Figure 1:**
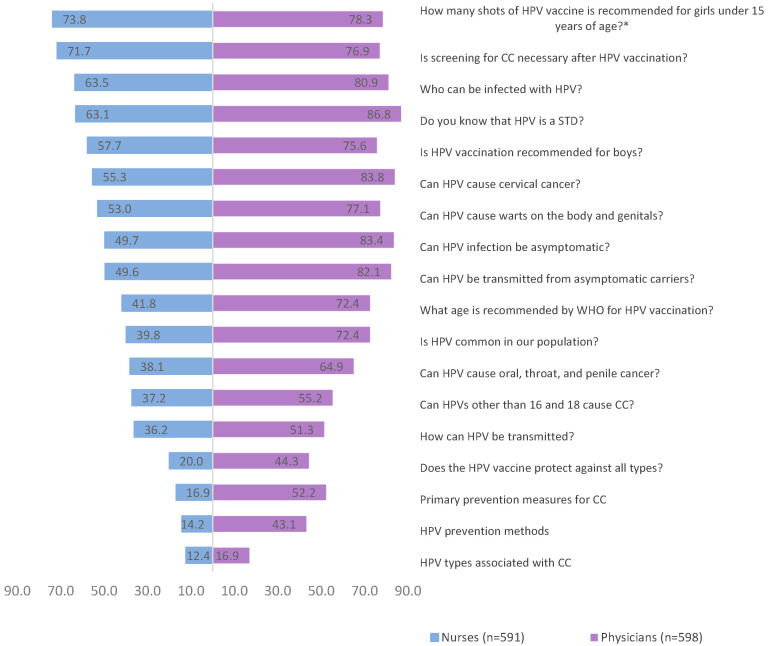
Comparison of correct answers between nurses and physicians, % (from Supplementary Table S2). * *p* > 0.05.

The distribution of correct answers was compared between nurses and physicians (Figure 1), and between healthcare professionals specialising in obstetrics and gynaecology and other specialties (Supplementary Table S1). On average, nurses demonstrated a percentage of correct answers of 44.1%, and physicians demonstrated a percentage of 66.5%. Healthcare professionals specialising in obstetrics and gynaecology showed the best knowledge, with an average percentage of correct answers to all questions of 70.9%, compared with 52.4% for other specialties. The most difficult questions for all groups were specific questions about types of HPV associated with cancer, prevention of HPV infection, primary prevention of CC, and routes of HPV transmission.

### 3.3. Factors Influencing Healthcare Professionals’ Knowledge of HPV and Vaccine Recommendation

Almost three-quarters of survey participants (72.6%) expressed a positive intention to recommend HPV vaccine to their patients.

Table 2 shows the multivariate logistic regression analysis models examining the association of higher knowledge and awareness of HPV and the HPV vaccine (≥11 out of 18) among healthcare professionals in Kazakhstan, and their positive intention to recommend the HPV vaccine to patients, with the study covariates. Higher knowledge scores were significantly associated with urban versus rural residence, higher income, higher education, specialisation in obstetrics and gynaecology, and more recent training in HPV knowledge and ongoing professional updates regarding HPV. In addition, higher knowledge scores were associated with positive attitudes towards childhood vaccination and with knowing people with cervical cancer. Factors such as gender, age group, and work experience were not significantly associated with higher knowledge.

A positive intention to recommend the HPV vaccine was significantly higher among those with higher HPV knowledge scores, those who continually updated their HPV knowledge, those familiar with cervical cancer, and those with positive attitudes towards the COVID-19 vaccine and childhood vaccination within the national schedule. Each knowledge point increased the likelihood of recommending the HPV vaccine by 43%. Professionals who constantly update their knowledge of HPV and the vaccine are almost three times more likely to recommend HPV vaccination than those who have not updated their knowledge for more than 10 years. Professionals in specialties other than general practice and obstetrics were less likely to recommend the HPV vaccine. A positive intention to recommend the vaccine was not associated with gender, age, place of residence, work experience, income, religion, or level of religiosity.

### 3.4. Healthcare Professionals’ Perception of Barriers to HPV Vaccine Uptake

**Figure 2:**
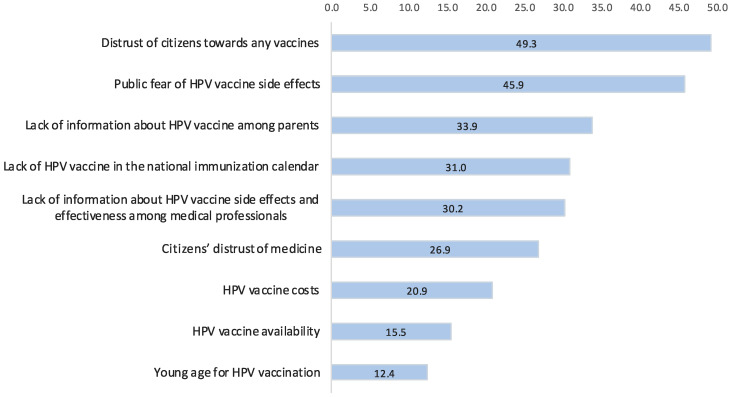
Barriers to HPV vaccination as reported by healthcare professionals in Kazakhstan (n = 1189), %.

As part of the survey of healthcare professionals, barriers to HPV vaccine uptake were investigated. Figure 2 presents data on the prevalence of these barriers among health professionals in Kazakhstan. The most important barrier, according to healthcare professionals, is the general mistrust of citizens towards all vaccines, reported by 49.4% of respondents. This was closely followed by public fear of HPV vaccine side effects, lack of information about the HPV vaccine among parents, and the absence of the HPV vaccine in the national immunisation schedule. At the time of the survey (2023), the HPV vaccine was not yet included in the national immunisation calendar in the Republic of Kazakhstan.

More than half of respondents (52.0%) reported difficulties in advising patients about HPV vaccination. Lack of information about the HPV vaccine, its safety profile, and effectiveness were the most common reasons for these difficulties. The unavailability of the vaccine was cited by 8.6% of healthcare professionals, and discomfort about discussing children’s sexual behaviour was cited by 5.8%.

### 3.5. HPV Vaccination Information Channels and Communication Practices Among Healthcare Professionals 

**Figure 3:**
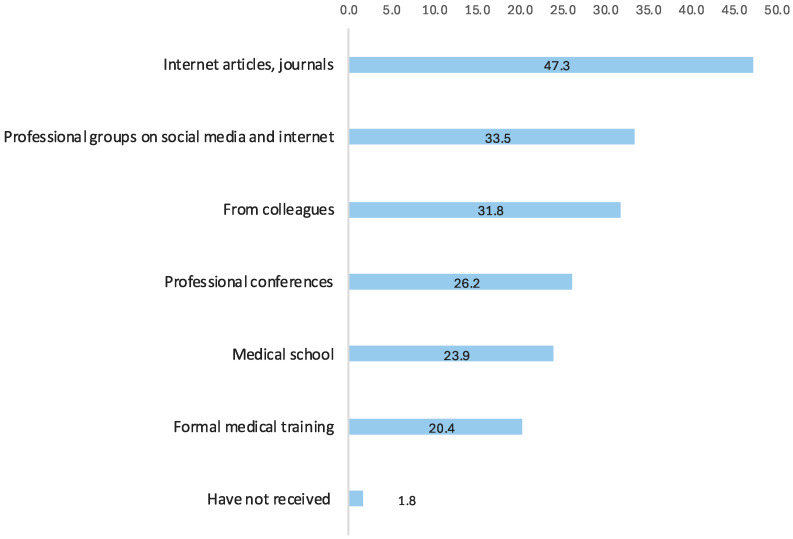
Sources used for acquiring and updating knowledge of HPV and HPV vaccines among healthcare professionals in Kazakhstan (n = 1189), %.

Among survey participants, 90.0% of healthcare professionals expressed a strong desire to improve their knowledge of HPV and the HPV vaccine, with 20.5% reporting that their last knowledge update was more than five years ago. Almost half cited internet journals and articles as their primary source of information, followed by professional groups on social media and online forums, and input from colleagues. Figure 3 illustrates the prevalence of various sources used for knowledge acquisition and updating among healthcare professionals in Kazakhstan.

Differences in sources of information about HPV and the HPV vaccine were observed between nurses and physicians and between allied health professionals with lower (<11.0) and higher (≥11.0) knowledge in Kazakhstan (Table 3 and Supplementary Table S3). The most commonly used sources of knowledge among nurses were internet journals and articles, followed by colleagues and social media. In contrast, physicians mainly relied on internet journals and articles, followed by social media, medical school, and professional conferences. Physicians generally used a wider range of academic and professional sources than nurses. Among those who received no information about HPV and the HPV vaccine, the proportion of nurses was more than double that of physicians (2.7% vs. 0.8%; *p* = 0.015).

An analysis of sources of information among healthcare professionals with lower (<11.0) and higher (≥11.0) HPV knowledge scores showed that those with higher scores were more likely to have received information from medical school (28.7% vs. 17.6%), formal training (26.2% vs. 12.8%), internet journals (55.8% vs. 36.2%), conferences (36.0% vs. 13.5%), and professional social media groups (42.1% vs. 22.2%) (Supplementary Table S3).

## 4. Discussion

Over the past 15 years, there has been an increase in the incidence of CC among Kazakhstani women, with the peak incidence shifting to a younger age group, from 50–55 years to 40–44 years [25]. Since 2008, Kazakhstan has implemented a national cytology-based screening programme for CC, with coverage rates varying from 46.2% in 2012 to 83.2% in 2019. Consequently, there has been a modest decline in mortality rates [26,27]. However, challenges remain, particularly with regard to HPV-based screening and vaccinating younger populations against HPV. Studies indicate that transitioning to HPV-based screening enhances programme effectiveness, while the introduction of self-testing improves access to screening, particularly for women in underserved areas, and could raise awareness about HPV [28,29]. The HPV vaccination programme in Kazakhstan was suspended four years after its introduction due to widespread parental refusal, spurred by extensive media coverage of side effects. Possible reasons for this refusal included low awareness and poor communication from health services to parents and adolescents, coupled with the dissemination of negative information in the media [12]. As is known based on a similar situation in Japan [30], this probably will increase the future burden of CC among girls born between 2005 and 2012 in Kazakhstan due to missed immunisation. In this study, we analysed the level of knowledge about HPV and the HPV vaccine, the desire to recommend the HPV vaccine, and perceived barriers to the introduction and implementation of HPV vaccination among healthcare professionals in the Republic of Kazakhstan. In particular, we analysed associations with demographic, social, and professional determinants.

### 4.1. Knowledge of HPV and the HPV Vaccine

In this survey, participants answered an average of 61.1% of questions about HPV and the HPV vaccine correctly, with the largest gaps being in specific advanced knowledge. Higher levels of knowledge were found among healthcare professionals with higher education and income, in urban areas, with more professional experience, from primary healthcare organisations, specialising in obstetrics and gynaecology, and those who had updated their HPV knowledge within the last five years.

Several studies have shown that knowledge levels vary by specialty, gender, work setting, working hours, and recent HPV education [31,32]. General practitioners, gynaecologists, and paediatricians showed higher awareness of HPV genotypes than other specialties, with knowledge differences observed between rural and urban practitioners [33]. In other studies, lower levels of knowledge were associated with male gender, fewer hours worked in a clinic, and smaller health centres [34]. A study of knowledge among health professionals in the United Kingdom found that more recent training correlated with higher levels of knowledge [35].

The main knowledge gaps in our study were in more advanced knowledge (oncogenic HPV genotypes, vaccination of boys, dosage regimen), while relatively general questions did not pose difficulties. Similarly, in all countries, up to 60–80% of professionals had a high level of knowledge about HPV infection, but lacked a detailed understanding of vaccine mechanisms and the benefits of HPV vaccination [31].

### 4.2. Intention to Recommend HPV Vaccination

In our study, 72.6% of health professionals were willing to recommend HPV vaccination for girls. This is consistent with other studies, where the average intention to recommend HPV vaccination was 66.9%. In these studies, increased rates of HPV vaccine recommendation were associated with higher levels of knowledge, national and professional guidelines on HPV vaccination, and beliefs in the efficacy and safety of the vaccine [32]. In our study, physicians with higher knowledge of HPV and the HPV vaccine, those specialising in obstetrics and gynaecology and general practice, and those with a positive attitude towards the COVID-19 vaccine were statistically significantly more likely to recommend HPV vaccination. However, this association was not observed in all groups; for example, specialists with two or more university degrees had higher knowledge but were less likely to recommend the vaccine. Some studies also suggest that knowledge of HPV and the HPV vaccine does not always lead to recommendation. Chawla et al. found that only 47% of participants would recommend HPV vaccination to young women, although 81% were aware of vaccines to prevent CC [33]. One explanation for low vaccination uptake may be a strong awareness of other methods of preventing cervical cancer, such as Pap smears [36]. However, most studies suggest that lack of information and knowledge about HPV and CC among health professionals is the main reason for low uptake of HPV vaccination programmes, especially among nurses [31].

In our study, physicians were more likely to recommend HPV vaccination than nurses. This is consistent with a study of COVID-19 vaccination, where nurses were significantly more likely to refuse vaccination [37]. In our survey, specialists were more likely than others involved in HPV vaccination (general practitioners, family physicians, and obstetricians/gynaecologists) to have a higher intention to recommend HPV vaccination, which may be a positive sign for a successful HPV vaccination programme. Factors such as gender, income level, level of religiosity, and work experience did not have a statistically significant association with intention to recommend HPV vaccination among health professionals in Kazakhstan.

### 4.3. Barriers to Recommending HPV Vaccination

In our study, health professionals identified several barriers to HPV vaccination in Kazakhstan, including citizens’ mistrust of all vaccines and fear of side effects. A significant number of participants cited a lack of information about the side effects and effectiveness of the HPV vaccine as a barrier to recommending it. In our study, mistrust of medicine was identified as a significant barrier to HPV vaccination, consistent with another study [38]. Participants identified low parental awareness of the HPV vaccine as a common barrier, similar to findings in the United States, where low parental education was identified as a barrier [39]. Trust in health professionals and strong recommendations from physicians are important factors in parents’ decisions to vaccinate their children against HPV [40].

Parental fear of side effects was another common barrier to HPV vaccination identified by health professionals. The HPV vaccine, no less than other types of vaccines, is surrounded by many misconceptions and fears, especially regarding its safety [16,41]. HPV vaccination in Kazakhstan was directly affected by these misconceptions, which ultimately led to the failure of the pilot programme in 2017 [12]. The challenge for health professionals is to address these misconceptions. Research during active vaccination against COVID-19 shows an increased prevalence of burnout among healthcare workers involved in vaccination, highlighting the need for psychological support [42]. In our study, about half of the health workers experienced difficulties in counselling about the HPV vaccine, often due to lack of information and less often due to its inaccessibility and concerns about its association with children’s sexual behaviour. A study of Ghanaian nurses found that the majority of unvaccinated participants cited lack of information about HPV vaccination as a factor in their decision [43]. Most participants in our study expressed a desire to improve their knowledge of the HPV vaccine, highlighting the need for increased education and awareness among healthcare professionals in Kazakhstan.

### 4.4. Communication and Sources of Information

Our study showed differences in information sources for acquiring and updating knowledge about HPV and the HPV vaccine between nurses and physicians, and between health professionals with different levels of knowledge. In our study, physicians and health professionals with higher levels of knowledge about HPV and the HPV vaccine appeared to use a wider range of information sources and to rely more on academic and professional channels than nurses and health professionals with lower levels of knowledge. Regardless of the group, a significant number of healthcare professionals actively engaged in professional communication with each other, including through social media platforms. Analysis of the sources of knowledge updates among healthcare professionals reveals a strong reliance on scientific literature and educational programmes offered by universities and other educational institutions. In addition, health professionals actively engage in professional communication through conferences and professional groups on social networks. In our study, formal professional education in medical schools and training programmes were cited as sources of information by 36.1% and 22.7% of physicians, respectively. In comparison, a higher proportion of physicians in the United States reported receiving information from professional organisations (50.0%) and the Advisory Council on Immunization Practices (36.0%). A similar proportion of physicians in both Kazakhstan and the United States reported using conferences as a source of information (36.0% and 33.1%, respectively). Consultation with colleagues was also a common source in both countries, with 29.4% of Kazakh physicians and 32.4% of American physicians relying on this method, which may reflect similarities in information-seeking behaviour. In contrast, the use of internet sites as a source of information was significantly higher in Kazakhstan (55.4%) than in the United States, where only 20.2% of obstetricians and gynaecologists reported using online resources. This difference may be due to the limited information available from professional organisations in Kazakhstan [44]. A study from Norway found that public health nurses were more likely to obtain knowledge from the Norwegian Institute of Public Health, while general practitioners were more likely to rely on professional journals and reference books. However, in our study, nurses were significantly less likely to use training programmes, because the HPV vaccination campaign had not yet started at the time of the study [45]. Globally, consultation with colleagues, reading journal articles, and using online resources such as Medline/PubMed are among the most commonly used methods by physicians for obtaining information. However, factors such as lack of time and inadequate search skills are commonly cited as barriers to accessing the information they need [46]. Mistrust of vaccination and medicine among citizens was one of the main barriers identified by healthcare professionals in our study, highlighting the need for communication training for healthcare workers on how to talk to vaccine-hesitant individuals. Studies show that regular training in communication, combined with other strategies, can effectively increase vaccination coverage [39,47]. Moreover, studies indicate that when physicians talk about HPV vaccination, their messages are often perceived as condescending, highlighting the need for specialised training in effective public communication [48].

#### Limitations

This study is the first in Kazakhstan to investigate the knowledge, awareness, and attitudes of health professionals regarding HPV and the HPV vaccine. However, several limitations must be acknowledged. This study was conducted at a time when the HPV vaccine was not available or introduced in the country. Therefore, knowledge and opinions may change as vaccination practices become more established. The use of a questionnaire and snowball sampling may have affected the representativeness of the sample. The involvement of medical organisations in the distribution of the questionnaires was intended to mitigate this limitation. In our study, the majority of healthcare professionals were female and urban residents, which may influence the representativeness of the sample due to the sampling method. However, the Bureau of Statistics of Kazakhstan reports an urban-to-rural population ratio of 1.7:1, and women account for 70–80% of healthcare workers in the country. While internet access is generally widespread in Kazakhstan, some areas still lack adequate access or face financial barriers, which may have excluded some participants. In our study, the survey was distributed via Google Forms across multiple digital platforms, including social media and email. The use of this online survey method made it impossible to determine the survey response rate, as the total number of individuals who received the survey link is unknown. Acknowledging this limitation, we selected this method to ensure broader reach. The study design does not allow causal relationships to be established between influencing factors and intention to recommend the HPV vaccine. In addition, the mix of simple and complex questions in the questionnaire may have influenced the overall responses, potentially biasing the results.

## 5. Conclusions

This study found that knowledge of HPV and the HPV vaccine among health professionals in Kazakhstan varied significantly by specialty, educational background, and other sociodemographic and professional characteristics. Higher levels of knowledge were associated with an increased likelihood of recommending HPV vaccination. These findings highlight the urgent need for a tailored, multifaceted communication strategy that addresses the diverse needs of health professionals in Kazakhstan, including by reducing inequalities in knowledge acquisition and increasing access to quality knowledge through medical education and continuing education opportunities for nurses, especially those involved in the vaccination process. Educational programmes for health professionals should include both medical school and specialised training to improve knowledge of HPV and the HPV vaccine. International public health organizations including WHO, UNICEF, GAVI, and others, provide essential support to healthcare professionals by offering resources, training, and public outreach. This collaboration enhances countries’ capacity to strengthen HPV prevention efforts and improve vaccination coverage, particularly in resource-limited settings. Access to reliable information should also be widened through the organisation of conferences, improved access to journals in the language most easily understood, and the development of professional websites with high-quality information on the HPV vaccine. The widespread use of social networks, as well as peer-to-peer communication among health professionals to gain knowledge, demonstrates the need to encourage experts to disseminate accurate information and facilitate discussions on these platforms about the upcoming introduction of HPV vaccination. A comprehensive approach should also include specialised training, not only to increase knowledge of HPV and the vaccine, but also to communicate with parents/guardians and adolescents. Future research should evaluate the effectiveness of these methods, while adapting evidence-based international practices to local conditions to improve the success of the HPV vaccination programme in Kazakhstan.

The results of this study can help inform the public health strategies and policies of the Republic of Kazakhstan, contribute to the development of educational and policy initiatives with targeted communication to prevent a repetition of previous negative experiences with HPV vaccination in Kazakhstan, more fully prepare and aid the development of a country-specific communication plan for the upcoming introduction of the HPV vaccine, and contribute to local and global efforts to eliminate cervical cancer and other HPV-associated diseases. In addition, improving communication strategies with the public to increase confidence in medical and preventive measures remains a major public health challenge. This requires taking into account local social and cultural characteristics to improve the uptake and implementation of vaccination programmes.

## Figures and Tables

**Table 1:** Sociodemographic data of study participants and HPV total knowledge score (median, IQR) (n = 1189).

Characteristics	n	%	Total Score, Me [Q1–Q3]	*p*-Value *
Age (Median, IQR)	37.0 [28.0–48.0]			
Gender				
Female	1045	87.9	11.0 [7.0–14.0]	0.234
Male	144	12.1	10.0 [7.0–14.0]	
Place of residence				
Rural	475	39.9	10.0 [6.0–12.0]	<0.001
Urban	714	60.1	11.0 [8.0–14.0]	
Occupation				
Nurses	591	49.7	9.0 [5.0–11.0]	<0.001
Physicians	598	51.3	13.0 [10.0–15.0]	
Work experience, years				
0–4	316	26.6	9.0 [5.0–13.0]	<0.001
5–10	230	19.3	11.0 [7.0–14.0]	
10–20	298	25.1	12.0 [9.0–14.0]	
More than 20 years	345	29	11.0 [8.0–14.0]	
Medical organization type				
Primary medicine organisation	799	67.2	11.0 [8.0–14.0]	<0.001
Inpatient medical organisation	390	32.8	10.0 [6.0–13.0]	
Specialty				
General practice	201	16.9	10.0 [6.0–13.0]	<0.001
Obstetrics and gynaecology	194	16.3	14.0 [11.0–16.0]	
Other specialties	794	66.8	10.0 [6.5–13.0]	
Would you recommend HPV vaccination to your patients?				
No	326	27.4	8.0 [4.0–11.0]	<0.001
Yes	863	72.6	12.0 [8.5–14.0]	
Total knowledge score, median [IQR]	11.0 [7.0–14.0]			

* Mann–Whitney U test and Kruskal–Wallis test were applied.

**Table 2:** Multivariate logistic regression analysis assessing factors associated with providers’ knowledge of HPV and the HPV vaccine and their recommendation of HPV vaccination (n = 1189).

Variables	Higher Knowledge Score (≥11) OR *; 95%CI	*p*-Value	Positive Intention to Recommend HPV Vaccination OR; 95%CI	*p*-Value
Age groups				
20–29 years old	Reference group		Reference group	
30–39 years old	0.983; 0.619–1.561	0.943	0.843; 0.536–1.325	0.459
40–49 years old	0.902; 0.553–1.471	0.679	0.856; 0.533–1.373	0.518
50–59 years old	0.968; 0.582–1.611	0.901	0.950; 0.572–1.578	0.844
60 and over	1.091; 0.479–2.484	0.835	0.710; 0.324–1.556	0.393
Gender				
Female	Reference		Reference	
Male	0.747; 0.478–1.169	0.202	0.866; 0.567–1.324	0.507
Place of residence				
Rural	Reference		Reference	
Urban	1.599; 1.161–2.201	0.004	0.875; 0.636–1.204	0.412
Monthly personal income				
Less than KZT 147.000 (less than USD 312)	Reference		Reference	
Over KZT 147.000 (over USD 312)	1.445; 1.045–1.999	0.026	0.811; 0.585–1.125	0.209
Education				
Nurses	Reference		Reference	
Physicians	4.484; 3.233–6.217	<0.001	1.111; 0.797–1.550	0.535
Religion				
Muslim	Reference		Reference	
Christian	2.038; 1.268–3.275	0.003	0.530; 0.344–0.818	0.004
Other	N/A**		N/A**	
Not religious	4.054; 1.960–8.387	<0.001	0.625; 0.336–1.162	0.137
Level of religiosity				
Not religious	Reference		Reference	
Not really religious	1.582; 0.899–2.783	0.112	1.637; 0.962–2.786	0.069
Moderately religious	2.360; 1.434–3.884	0.001	1.151; 0.729–1.819	0.546
Quite religious	1.864; 1.052–3.302	0.033	1.364; 0.797–2.334	0.257
Very religious	1.357; 0.608–3.028	0.456	1.441; 0.662–3.138	0.358
Work experience. years				
<5 years	Reference		Reference	
≥5 years	1.428; 0.927–2.199	0.106	0.846; 0.554–1.294	0.441
Specialty				
General practice	Reference		Reference	
Obstetrics–gynaecology	4.221; 2.325–7.664	<0.001	0.994; 0.566–1.744	0.982
Other specialties	1.080; 0.729–1.601	0.701	0.588; 0.391–0.885	0.011
Time since last HPV and HPV vaccine knowledge update				
More than 10 years ago	Reference		Reference	
5–10 years ago	0.911; 0.481–1.725	0.775	1.582; 0.888–2.819	0.120
Up to 5 years	2.250; 1.413–3.583	0.001	1.602; 1.057–2.427	0.026
Update constantly	2.682; 1.609–4.471	<0.001	3.202; 1.966–5.213	<0.001
Attitudes to COVID-19 vaccination				
Negative/Doubtful	Reference		Reference	
Positive	1.157; 0.838–1.597	0.377	2.276; 1.674–3.096	<0.001
Children National Vaccination Program attitude				
Negative/Doubtful	Reference		Reference	
Positive	2.695; 1.924–3.773	<0.001	1.462; 1.053–2.030	0.023
HPV and HPV vaccine knowledge				
Lower (total score ≤ 11.0)	N/A**		Reference	
Higher (total score > 11.0)	N/A**		1.823; 1.304–2.547	<0.001
Having familiar people with cervical cancer				
No	Reference		Reference	
Yes	2.206; 1.631–2.984	<0.001	2.040; 1.504–2.767	<0.001

* OR; 95%CI—odds ratio with 95% confidence interval. ** N/A: Not Applicable.

**Table 3:** Comparative analysis of sources of information about HPV and HPV vaccine used by nurses and physicians in Kazakhstan (n = 1189).

	Nurses (n = 591)		Physicians (n = 598)		Test of Difference	*p*-Value
Information source	n	%	n	%	χ^2^	
Internet articles, journals	231	39.1	331	55.4	31.55	<0.001
Colleagues	202	34.2	176	29.4	3.09	0.081
Professional groups on social media	164	27.7	234	39.1	17.29	<0.001
Formal medical training	106	17.9	136	22.7	4.24	0.044
Professional conferences	97	16.4	215	36.0	58.64	<0.001
Medical school	68	11.5	216	36.1	99.06	<0.001
Have not received	16	2.7	5	0.8	6.0	0.015
Other	3	0.5	1	0.2	1.03	0.371

## Data Availability

The datasets used and analysed in the current study are available from the corresponding author on reasonable request.

## Supplementary Materials

The following supporting information can be downloaded at https://www.mdpi.com/article/10.3390/vaccines12111225/s1, Table S1: Distribution of correct answers on questions about HPV and HPV vaccine between healthcare professionals of Non- Obstetrician -Gynecologist and Obstetrician -Gynecologist specialty; Table S2: Distribution of right answers on questions about HPV and HPV vaccine between healthcare professionals of Nurses and Physicians; Table S3: Comparison of sources of information about HPV and HPV vaccine among healthcare professionals with low (<11.0) and high (≥11.0) knowledge (n = 1189).
