# Supplementary material for: Developing HPV Vaccination Communication Strategies: Assessing Knowledge, Attitudes, and Barriers Among Healthcare Professionals in Kazakhstan
Source: Vaccines (Basel). 2024 Oct 28;12(11):1225. doi: 10.3390/vaccines12111225 (PMC11598784; doi:10.3390/vaccines12111225)
Supplement: Supplementary file 1 [file vaccines-12-01225-s001.zip › vaccines-3248613-supplementary.pdf]

Supplementary

**Table S1 – Distribution of correct answers on questions about HPV and HPV vaccine between healthcare professionals of Non- Obstetrician -Gynecologist and Obstetrician -Gynecologist specialty**

| | Question on HPV and HPV knowledge (correct answers) | Non- Obstetrician - Gynecologist (995) | | Obstetrician - Gynecologist (194) | | Test of difference | |
|---|---|---|---|---|---|---|---|
| | | n | % | n | % | χ2 | p-value |
| **Q1** | Do you know that HPV is a sexually transmitted infection? | 717 | 72.1 | 175 | 90.2 | 28.526 | <0.001 |
| **Q2** | Can HPV cause warts on the body and genitals? | 616 | 61.9 | 158 | 81.4 | 27.3 | <0.001 |
| **Q3** | Can HPV cause cervical cancer? | 663 | 66.6 | 165 | 85.1 | 26.0 | <0.001 |
| **Q4** | Can HPV cause oral, throat, and penile cancer? | 485 | 48.7 | 128 | 66.0 | 19.3 | <0.001 |
| **Q5** | Who can be infected with HPV? | 692 | 69.5 | 167 | 86.1 | 22.1 | <0.001 |
| **Q6** | Can HPV infection be asymptomatic? | 627 | 63.0 | 166 | 85.6 | 37.2 | <0.001 |
| **Q7** | Can HPV be transmitted from asymptomatic carriers? | 627 | 63.0 | 157 | 80.9 | 23.2 | <0.001 |
| **Q8** | Is HPV common in our population? | 514 | 51.7 | 154 | 79.4 | 50.7 | <0.001 |
| **Q9** | How can HPV be transmitted? | 392 | 39.4 | 129 | 66.5 | 48.4 | <0.001 |
| **Q10** | Primary prevention measures for CC | 303 | 30.5 | 109 | 56.2 | 47.5 | <0.001 |
| **Q11** | HPV types associated with CC | 119 | 12.0 | 55 | 28.4 | 34.9 | <0.001 |
| **Q12** | Can HPVs other than 16 and 18 cause CC? | 424 | 42.6 | 126 | 64.9 | 32.6 | <0.001 |
| **Q13** | HPV prevention methods | 244 | 24.5 | 98 | 50.5 | 53.5 | <0.001 |
| **Q14** | Does the HPV vaccine protect against all types? | 287 | 28.8 | 96 | 49.5 | 31.7 | <0.001 |
| **Q15** | What age is recommended by WHO for HPV vaccination? | 530 | 53.3 | 150 | 77.3 | 38.4 | <0.001 |
| **Q16** | How many shots is recommended by WHO for girls under 15 years of age? | 761 | 76.5 | 143 | 73.7 | 0.68 | 0.408 |
| **Q17** | Is screening for CC necessary after HPV vaccination? | 729 | 73.3 | 155 | 79.9 | 3.7 | 0.053 |
| **Q18** | Is HPV vaccination recommended for boys? | 650 | 65.3 | 143 | 73.7 | 5.1 | 0.023 |

**Table S2 – Distribution of right answers on questions about HPV and HPV vaccine between healthcare professionals of Nurses and Physicians**

| | Question on HPV and HPV knowledge (correct answers) | Nurses (591) | | Physicians (598) | | Test of difference | |
|---|---|---|---|---|---|---|---|
| | | n | % | n | % | χ2 | p-value |
| **Q1** | Do you know that HPV is a sexually transmitted infection? | 373 | 63.1 | 519 | 86.8 | 88.9 | <0.001 |
| **Q2** | Can HPV cause warts on the body and genitals? | 313 | 53.0 | 461 | 77.1 | 76.2 | <0.001 |
| **Q3** | Can HPV cause cervical cancer? | 327 | 55.3 | 501 | 83.8 | 113.8 | <0.001 |
| **Q4** | Can HPV cause oral, throat, and penile cancer? | 225 | 38.1 | 388 | 64.9 | 85.6 | <0.001 |
| **Q5** | Who can be infected with HPV? | 375 | 63.5 | 484 | 80.9 | 45.3 | <0.001 |
| **Q6** | Can HPV infection be asymptomatic? | 294 | 49.7 | 499 | 83.4 | 152.0 | <0.001 |
| **Q7** | Can HPV be transmitted from asymptomatic carriers? | 293 | 49.6 | 491 | 82.1 | 140.0 | <0.001 |
| **Q8** | Is HPV common in our population? | 235 | 39.8 | 433 | 72.4 | 128.7 | <0.001 |
| **Q9** | How can HPV be transmitted? | 214 | 36.2 | 307 | 51.3 | 27.6 | <0.001 |
| **Q10** | Primary prevention measures for CC | 100 | 16.9 | 312 | 52.2 | 163.1 | <0.001 |
| **Q11** | HPV types associated with CC | 73 | 12.4 | 101 | 16.9 | 4.9 | 0.027 |
| **Q12** | Can HPVs other than 16 and 18 cause CC? | 220 | 37.2 | 330 | 55.2 | 38.6 | <0.001 |
| **Q13** | HPV prevention methods | 84 | 14.2 | 258 | 43.1 | 121.4 | <0.001 |
| **Q14** | Does the HPV vaccine protect against all types? | 118 | 20.0 | 265 | 44.3 | 80.7 | <0.001 |
| **Q15** | What age is recommended by WHO for HPV vaccination? | 247 | 41.8 | 433 | 72.4 | 113.8 | <0.001 |
| **Q16** | How many shots is recommended by WHO for girls under 15 years of age? | 436 | 73.8 | 468 | 78.3 | 3.3 | 0,07 |
| **Q17** | Is screening for CC necessary after HPV vaccination? | 424 | 71.7 | 460 | 76.9 | 4.2 | 0.04 |
| **Q18** | Is HPV vaccination recommended for boys? | 341 | 57.7 | 452 | 75.6 | 42.8 | <0.001 |

**Table S3 - Comparison of sources of information about HPV and HPV vaccine among healthcare professionals with low (<11.0) and high (≥11.0) knowledge (n=1189)**

| | Lower Knowledge (n=517) | | Higher Knowledge (n=672) | | Test of difference | p-value |
|---|---|---|---|---|---|---|
| Information sources | n | (%) | n | (%) | χ2 | |
| Internet articles, journals | 187 | 36.2 | 375 | 55.8 | 45,19 | <0,001 |
| Colleagues | 157 | 30.4 | 221 | 32.9 | 0,86 | 0,355 |
| Professional groups on social media and internet | 115 | 22.2 | 283 | 42.1 | 51,80 | <0,001 |
| Professional conferences | 70 | 13.5 | 242 | 36.0 | 76,24 | <0,001 |
| Medical school | 91 | 17.6 | 193 | 28.7 | 19,87 | <0,001 |
| Formal medical training | 66 | 12.8 | 176 | 26.2 | 32,48 | <0,001 |
| Have not received | 21 | 4.1 | 0 | 0.0 | 27,79 | <0,001 |
| Other | 3 | 0.6 | 1 | 0.1 | 1,62 | 0,203 |
